# The Nonlinear Effects of Environmental Regulation on Ecological Efficiency of Animal Husbandry—Case Study of China

**DOI:** 10.3390/ani15081167

**Published:** 2025-04-18

**Authors:** Liyuan Shang, Jinhui Ning, Gaofei Yin, Wenchao Li, Juanjuan Wu, Cha Cui, Ruimei Wang

**Affiliations:** 1 College of Economics and Management, Hebei Agricultural University, Baoding 071000, China; b20233110921@cau.edu.cn (L.S.); ningjinhui1120@163.com (J.N.); 20247010671@pgs.hebau.edu.cn (J.W.); 2 College of Economics and Management, China Agricultural University, Beijing 100083, China; 3 State Key Laboratory of North China Crop Improvement and Regulation, Hebei Province Key Laboratory for Farmland Eco-Environment, College of Resources and Environmental Sciences, Hebei Agricultural University, Baoding 071000, China; pangpanggf@hebau.edu.cn (G.Y.); dachao279@hebau.edu.cn (W.L.)

**Keywords:** environmental regulation, ecological efficiency, space overflow, nonlinear effects, regional heterogeneity

## Abstract

Developed countries with animal husbandry are confronted with the pressing issues of ensuring stable livestock product supplies while maintaining ecological sustainability. Additional research is required to ascertain whether environmental regulation can effectively facilitate the green transformation of animal husbandry and establish a harmonious equilibrium between environmental protection and economic growth. It is essential for the empirical development of environmental policies in animal husbandry, as it evaluates the impact of regulatory measures on this sector’s ecological efficiency and precisely investigates the underlying mechanisms of these effects. This paper evaluates the nonlinear impact of environmental regulation policies on the ecological efficiency of animal husbandry using the super-efficiency EBM model, spatial Durbin model, and panel threshold model, which are based on panel data from 31 Chinese provinces (2010–2022). The findings indicated that: (1) The ecological efficiency and environmental regulation intensity of animal husbandry in China exhibited a fluctuating upward trend. The environmental regulation is ranked from high to low in the following order: Northeast, West, Central, and Eastern regions. Conversely, the regions with high ecological efficiency are concentrated in the Northeast and Western regions. (2) The impacts of environmental regulation on the ecological efficiency of animal husbandry were N-type nonlinear, with the extreme points being 6.322 and 9.456. Environmental regulation also produced an “inverted N” type spatial spillover effect on the ecological efficiency of animal husbandry in adjacent areas, with extreme values of 5.330 and 7.670. (3) Environmental regulation considerably enhanced the ecological efficiency of animal husbandry in the Eastern and Central regions in terms of location characteristics. The influence on the Western and Northeastern regions exhibited N-type nonlinear characteristics. (4) From 2017 to 2022, ER had an N-type nonlinear effect on animal husbandry ecological efficiency in terms of temporal heterogeneity. However, the effect was not significant from 2010 to 2016.

## Simple Summary

The impact of environmental regulations on the ecological efficiency of animal husbandry is complex and uncertain, and blindly implementing environmental policies is not a long-term solution for the sustainable development of animal husbandry. It is urgent to open the black box between environmental regulations and ecological efficiency, and to explore how policies can guide animal husbandry to maintain a balance between economic and environmental benefits, thereby promoting sustainable development of animal husbandry. This research evaluates the nonlinear impact of environmental regulation policies on the ecological efficiency of animal husbandry in China from 2010 to 2022. It was found that the impacts of environmental regulation on the ecological efficiency of animal husbandry are N-type nonlinear. Meanwhile, there are differences in the impact of environmental regulations on ecological efficiency in different regions, highlighting the need to implement region-specific policies.

## 1. Introduction

The demand for high-quality meat, eggs, and milk can be satisfied through animal husbandry. It is essential to global agricultural production. However, the accelerated advancement of animal husbandry has not only improved efficiency and economic benefits, but has also exacerbated environmental pollution concerns. The Food and Agriculture Organization of the United Nations (FAO) reported in 2013 that the livestock supply chain accounted for 14.5% of global greenhouse gas emissions [1]. Furthermore, the sustainable development of global agriculture was significantly impeded by the presence of nitrogen, phosphorus, and heavy metals in livestock manure, which not only contaminated water and soil resources but also posed health dangers to humans [2]. It is an imperative matter that must be resolved in order to improve the quality and quantity of animal products in all developed countries in the sector by balancing the stable supply of animal husbandry with the reduction in environmental pollution.

In order to enhance the economic efficiency of farming and mitigate environmental pollution, all countries have implemented a variety of ecological and environmental policies, including the Environmental Quality Incentive Program (EQIP) in the United States and the Agricultural Environment Program (AES) in the European Union. The Chinese government has implemented environmental regulation (ER) policies in response to the development requirements of ecological agriculture. These policies are dedicated to the transformation of animal husbandry and the attainment of sustainable development.

The first agricultural environmental protection law at the national level was the “Regulations on the Prevention and Control of Pollution from Large-Scale Livestock and Poultry Breeding” that were enacted in 2014. Several policy documents have been issued since that time, such as the “Water Pollution Prevention and Control Action Plan”, “Implementation Opinions on Accelerating the Resource Utilization of Livestock and Poultry Breeding Waste”, and “Opinions on Promoting High-Quality Development of Animal Husbandry”. These policies have significantly increased environmental protection pressures on local governments and livestock producers. According to data from two national pollution censuses, the emission intensities of chemical oxygen demand (COD), total nitrogen (TN), and total phosphorus (TP) per unit of livestock and poultry during the second national pollution census period (2017) decreased by 55.5%, 67.2%, and 57.9%, respectively, compared to the first national pollution census period (2007). Initially, related policies were centered on mandatory punitive measures, but they progressively evolved to incorporate management and technical policies. Policies were implemented to alleviate regional environmental pressure and foster high-quality development in the animal husbandry sector, including adjusting the layout of the livestock industry, promoting comprehensive utilization and harmless treatment of waste, and implementing ecological protection compensation [3,4].

Still, the implementation of ER policies is a two-sided process. On the one hand, these policies motivate breeding entities to implement practices that promote the introduction of high-quality livestock breeds, the reduction in feed cereal consumption, and the provision of facilities for the treatment of livestock waste, thereby establishing an ecological circular farming model. Conversely, the government’s excessive emphasis on ER may result in an overemphasis on environmental quality, underscoring the problem of a universal approach to environmental protection. This has the potential to increase production costs in the livestock sector and reduce economic benefits [5]. Therefore, the consequences are intricate and unpredictable; the mere enforcement of these regulations is not a sustainable long-term approach to animal husbandry. It is imperative to isolate specific environmental policies from a variety of influencing factors in order to elucidate the relationship between ecological efficiency and ER. It is essential to scientifically evaluate the impact of ER on the ecological efficiency of livestock husbandry in order to identify execution issues and evaluate the effectiveness of policy implementation. This study analyzed the nonlinear influence and spatial spillover effect of environmental regulation on animal husbandry eco-efficiency in China from 2010 to 2022, using a constructed environmental regulation intensity index and combining STIRPAT theory framework. This will help provide a scientific basis for policy effectiveness evaluation and implementation optimization, and promote animal husbandry to achieve a dynamic balance between ecological protection and production supply.

## 2. Literature Review and Hypothesis Development

### 2.1. Literature Review

#### 2.1.1. Relevant Research on ER

The primary focus of ER research is the measurement and evaluation of the impact of environmental regulatory policies. One method of measuring ER is to employ the quantity of environmental legislation as a metric [6]. Factor analysis and entropy weight are employed to quantify comprehensive environmental policy indices in accordance with the number of policies [7]. Another method entails the utilization of subjective evaluations from breeding producers to assess the severity of ER. Zhu et al. (2021) [8] employed micro-survey data to assess the perceptions of large-scale producers regarding the guiding, constraining, and incentivizing effects of ER. Furthermore, the measurement of ER is conducted using methods such as Partial Least Squares Regression (PLSR) and composite index methods, which employ external macro indicators such as pollutant emissions, economic development levels, and environmental governance costs [9,10,11].

The initial step in assessing the efficacy of ER is to evaluate the policies’ overall impact. Wei et al. (2021) [12] suggested that the objectives of pollution prevention policies in China’s animal husbandry had become increasingly refined and explicit; however, challenges such as inconsistent policy statements and inadequate policy implementation continue to exist. Furthermore, additional research demonstrated that the livestock sector’s vertical integration within the livestock industry chain was considerably enhanced by robust ER [13]. The second component emphasizes the influence of policies on particular demographics. According to research, farmers’ attitudes toward environmental regulation may potentially motivate them to implement sustainable production technologies [14].

#### 2.1.2. Research on the Ecological Efficiency of Animal Husbandry

Ecological efficiency (EE) assesses the integrated advancement of the economy and the environment, functioning as an essential instrument for appraising industrial sustainable growth [15]. Current research predominantly emphasizes calculation, attributes, and affecting factors. The principal methodologies encompass Stochastic Frontier Analysis (SFA), Data Envelopment Analysis (DEA), and their enhanced variants [16,17]. The studies included undesirable outputs such as carbon emissions from livestock farming, manure discharge, and water pollution in the indicator system [18], and characterized ecological efficiency as the overall technical efficiency constrained by environmental costs [19]. It is crucial to acknowledge that agricultural land and grasslands may assimilate livestock waste, resulting in measurable decreases in environmental pollution. Consequently, the absorption capacity must not be disregarded while assessing EE. Utilizing kernel density estimation and spatial Markov chains, this study examined the dynamic evolution and convergence patterns of EE from the standpoint of spatiotemporal evolution characteristics [20]. Research demonstrated that the energy efficiency of China’s cattle sector displayed a variable rising trajectory, characterized by a stepwise regional dispersion [19]. Elements including farmers’ per capita income, environmental governance, and agricultural financial assistance substantially impacted the EE of the livestock sector [18].

#### 2.1.3. Research on the Impact of ER on EE of Animal Husbandry

The research examines three facets of the influence of ER on the EE of animal husbandry. The studies mostly investigate the linear impacts of different environmental regulation types or individual environmental policies on animal husbandry and overall agricultural environmental efficiency. Research indicated that the “grass-animal balance” policy produced a positive linear influence on the economic efficiency of grassland animal husbandry [21]. Voluntary environmental regulation exerted a beneficial linear influence on-farm energy efficiency [22]. Secondly, the majority of current research has investigated the nonlinear effects of environmental restrictions on sustainable agricultural output through the nonlinear panel threshold model [11]. Researchers in animal husbandry have examined the nonlinear effects of industrial agglomeration on the efficiency of animal husbandry [23]. Thirdly, ER demonstrates a spatial spillover effect; specifically, ER policies can impact pollution prevention and management in both local and adjacent regions [24,25]. Most previous studies concentrate on the direct impact of ER policies on diminishing pollutant emissions from local animal husbandry, although there is a deficiency of research about the cross-regional effects of these policies. [5,21].

The relevant literature has established a robust foundation for the current study; nonetheless, there exists a possibility for additional exploration in the subsequent areas. Initially, environmental regulations (ER) are assessed indirectly through proxy indicators, primarily the degree of economic development. Additionally, some incorporate a number of environmental policies, which complicates the accurate reflection of efficiency disparities across various regions and policies, necessitating enhanced precision. Secondly, in the selection of unconventional indices for animal husbandry environmental efficiency, it is challenging to accurately represent the actual production conditions by disregarding the impact of the environment on livestock waste. The influence of ER on the EE of animal husbandry requires further elucidation. The existing research findings predominantly rely on linear regression; however, the nonlinear effects of environmental regulations on the eco-efficiency of animal husbandry require further validation. Fourthly, current research predominantly examines the influence of ecological reserves on the environmental efficiency of animal husbandry in the region, neglecting the spatial correlation and heterogeneity. The spatial attributes of the influence of ERs on the EE of animal husbandry require additional investigation.

To address the mentioned research gap, this study aims to achieve many objectives in this study: Initially, assess the EE intensity index based on policy efficacy and magnitude to precisely determine the intensity of environmental regulation. Secondly, this study thoroughly evaluates the carbon sink potential of manure absorption and feed grain production, and accurately represents the ecological efficiency value of animal husbandry in a more scientific manner. Thirdly, the nonlinear effects of environmental regulation on animal husbandry energy efficiency were examined in conjunction with the STIRPAT theoretical framework. Finally, the nonlinear spatial spillover effect of environmental regulation on animal husbandry energy efficiency was examined, taking into account the impact of geographical area.

### 2.2. Theoretical Basis and Research Hypothesis

**Figure 1:**
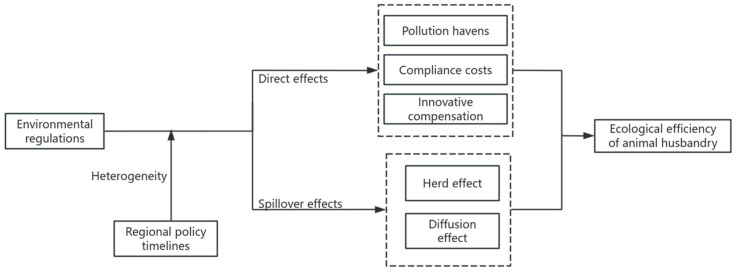
Mechanism of ER on EE of animal husbandry.

ER is a significant institutional framework that fosters the green and sustainable advancement of animal husbandry; yet, the impact of ER on EE in this sector remains ambiguous. Simultaneously, environmental management can overcome geographical constraints, and its influence on the EE may extend to adjacent regions, resulting in a spatial spillover effect. This research establishes a theoretical framework for the influence of ER on the EE of animal husbandry, as shown in Figure 1.

#### 2.2.1. Impact of ER on EE of Animal Husbandry

Current research often examines the influence of Environmental Regulation (ER) on Environmental Efficiency (EE) through the interplay of the “compliance cost” effect and the “innovation compensation” effect; however, the “pollution paradise” effect may also significantly affect EE. This paper examines the influence of ER on the EE of animal husbandry throughout three stages.

The first is the preliminary phase of environmental control. Currently, the ER policy is insufficient to induce cost alterations in the region’s livestock enterprises; nonetheless, the region may entice the livestock industry to transform into a “pollution paradise” by capitalizing on the low ER levels, thereby fostering the advancement of animal husbandry. The second phase is the development stage of environmental control. Elevating pollution control expenses and investments in green technology might augment the production burden on livestock and poultry farmers, leading to a “compliance cost” impact [26,27], thus diminishing the energy efficiency of animal husbandry in the near run. The third stage is the advanced phase of environmental regulation. Encouraging producers to implement clean and low-energy technologies can optimize factor allocation, diminish pollution emissions from livestock and poultry, enhance product value, and create an “innovation compensation” effect [28,29,30], thereby counteracting the “cost of compliance” effect and facilitating harmonious ecological and economic development [28]. Industrial technological innovation and sustainable development are influenced by numerous elements, and the impact of ER is not boundless; so, as regulatory intensity escalates, the “innovation compensation” effect shows diminishing marginal returns. In conclusion, Hypothesis 1 (H1) is proposed.

**H1:**  *ER has a nonlinear impact on animal husbandry’s EE, and the two have an “N” type relationship.*

#### 2.2.2. Spatial Spillover Effect of ER on Animal Husbandry EE

According to Tobler’s first geographical law, geographical things have a spatial correlation in spatial distribution, and local social and economic activities can influence the development of nearby areas, resulting in the “spatial spillover effect” [31,32]. Carbon and manure emissions from animal husbandry have externality characteristics, and the degree and stage characteristics of environmental regulation vary by region, so the intensity of environmental regulation may have spillover effects on the EE of animal husbandry in neighboring regions. Specifically, it includes two aspects:

First, consider the in-group impact. Resource constraints in neighboring areas are similar, and improving local environmental regulation levels will increase the importance of animal husbandry in neighboring areas [19], resulting in the synchronous development of an “economy-environment” with neighboring areas and the formation of an “in-group effect”. The second phenomenon is the diffusion effect. The applicable ecological technologies, production modes, and animal husbandry experiences can achieve geographical spillover through learning and imitation across regions. At the same time, animal husbandry with relatively high pollution may be transferred and spread to places with low environmental control intensity, with nearby areas being the continuation of polluting businesses [20]. In summary, Hypothesis 2 (H2) is proposed.

**H2:**  *There is a spatial spillover impact of ER on the EE of animal husbandry in neighboring areas.*

#### 2.2.3. Heterogeneity of Impact of Environmental Regulations on Animal Husbandry EE

China has a wide territory, and the economic endowment and natural environment of animal husbandry vary greatly between regions [20]. The eastern region’s quick economic development and strong technological innovation capacity allow for the more efficient implementation of various environmental protection measures in animal husbandry. The central region is an important animal husbandry production area, but the resource endowment is restricted, ecological pressure is high, and technological innovation ability is low. The resource endowment for animal husbandry in western and northeast China is relatively abundant, but the economic foundation and environmental protection technologies are relatively dated, and the ecological environment is vulnerable. As a result of environmental pressure, economic base, and other factors, environmental regulatory regulations are implemented differently across regions [2,5], potentially having a regional impact on the economic and environmental benefits of animal husbandry. In summary, Hypothesis 3 (H3) is proposed.

**H3:**  *Environmental regulation’s impact on animal husbandry varies by area.*

China’s animal husbandry policies are always being optimized and adjusted to reflect the industry’s growth, and the methods used to implement these policies vary substantially among stages. In times of inadequate livestock supply, animal husbandry policies emphasize enhancing productivity and revenue. The government supports breeding scale expansion through financial subsidies, technical support, infrastructure construction, and other measures [12]. However, as the scale of animal husbandry grows, so does the ecological environment pressure caused by negative externalities, and the policy goal of animal husbandry is shifting toward green, efficient, and sustainable [5,20]. The government has promoted the adjustment of livestock production methods by establishing strict environmental protection standards, promoting green production technologies, and promoting county-wide projects for the utilization of livestock and poultry waste resources, all of which may have an impact on livestock production EE. In summary, Hypothesis 4 (H4) is proposed.

**H4:**  *Environmental rules’ impact on animal husbandry EE varies throughout time.*

## 3. Materials and Methods

### 3.1. Research Framework

**Figure 2:**
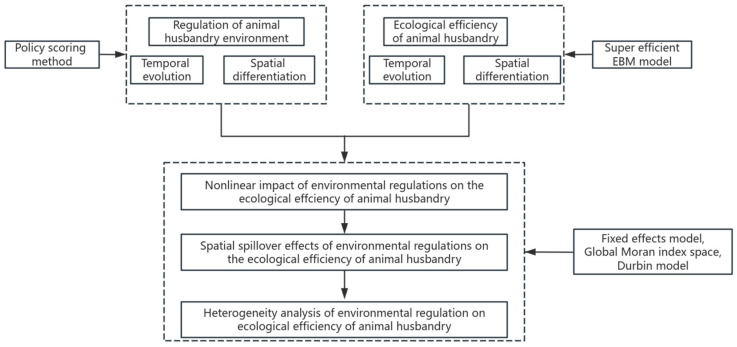
Research framework.

This paper develops a unique research approach to the impact of ER on animal husbandry EE, which includes both the nonlinear impact of ER on animal husbandry EE and the geographical spillover effect on animal husbandry EE in nearby areas. Among these, environmental regulation takes into account policy effectiveness and level, and measures it using the policy scoring system. Animal husbandry EE evaluation indicators include the animal husbandry net carbon sink index and the animal manure emission intensity index, which indicate the environmental impact of animal waste consumption. The level of animal husbandry EE is determined using the super-efficiency EBM model (Figure 2).

### 3.2. Variable Selection and Measurement

#### 3.2.1. Measurement of ER

Based on the studies of Wang et al. (2023) [33] and Huan et al. (2024) [34], this paper measured ER in animal husbandry based on the number of environmental policies in animal husbandry, taking into account provincial heterogeneity and policy effectiveness, with the goal of improving the accuracy of environmental regulation measurement. First, the “Peking University Magic Weapon” database was searched for legislation and regulations governing animal husbandry and pollution management using keywords. Second, laws and regulations are categorized based on policy-issuing units and policy efficacy levels. Third, as shown in Table 1, assess the subject of policy issuance (national, ministerial, or provincial) as well as policy kinds (laws, regulations, rules, normative documents, and working documents), and score each policy based on its efficacy. Finally, the policy rating table calculates the weighted value as the ER intensity.

#### 3.2.2. Measurement of Animal Husbandry EE

(1) Index selection

Animal husbandry EE is the overall efficiency of measuring the input of production materials, economic output, and undesirable output in the process of animal husbandry production. This research develops an index system for animal husbandry ecological efficiency (AEC), as indicated in Table 2.

The input index, based on the Cobb–Douglas (CD) production function, includes the input of technology and feed supplies while taking labor and capital components into account, so more accurately reflecting the input of the animal husbandry ecological economy system. The predicted output index measures the economic advantage of animal husbandry based on its output value.

Carbon emissions from animal husbandry in the net carbon sink index are primarily associated with feed grains (planting, transportation, and processing), livestock breeding (intestinal fermentation, manure management, and feeding energy consumption), and livestock slaughtering and processing. Carbon sinks in animal husbandry mostly include the carbon absorption process in feed grain planting. Refer to relevant scholars’ carbon emission and sink coefficients, as well as their calculating methodologies. The load of manure in livestock emission intensity is assessed by livestock and poultry manure-bearing capacity per unit cultivated land area, with 45 tons/hectare being the theoretical maximum appropriate pollutant-carrying capacity of cultivated land [35].

**Table 2:** EE evaluation index system of animal husbandry.

Index Class	Index Selection	Index Calculation	Reference
Put into	Animal husbandry practitioners	Number of employees in agriculture, forestry and fishery × (output value of animal husbandry/total output value of Agriculture, forestry and fishery)	Han et al. (2020) [18]
	Animal husbandry machinery technology input	Total power of agriculture, forestry and fishery machinery × (output value of animal husbandry/total output value of Agriculture, forestry and fishery)	Han et al. (2020) [18]
	Investment in fixed assets in animal husbandry	Fixed investment in agriculture, Forestry, animal husbandry and fishery × (output value of animal husbandry/total output value of Agriculture, forestry, animal husbandry and fishery)	Han et al. (2020) [18]
	Livestock feed input	Output value of livestock and poultry feed (2010 base period, using price index deflator)	Bai et al. (2018) [36]
Expected output	Output value of animal husbandry	Using 2010 as the base period, the price index deflator is used	Han et al. (2020) [18]
Undesirable output	Net carbon sink index of animal husbandry	Livestock carbon emissions/livestock carbon sinks (based on LCA)	Tian and Zhang (2013) [37]
	Emission intensity of livestock and poultry manure	Livestock and poultry manure soil load/theoretical maximum suitable pollutant-bearing capacity of cultivated land	Zhang et al. (2020) [35]

(2) Animal Husbandry EE Measurement Model

Tone and Tsutsui (2010) [38] proposed the EBM model to address the issue of non-radial relaxation variables, making measurement findings more accurate. As a result, in this research, the super-efficiency EBM model with undesirable output is utilized to calculate the EE of animal husbandry, as shown in Formulas (1) and (2).

(1) $$\gamma *=\min\frac{\theta -\varepsilon^{-}\sum_{i=1}^{m}\frac{w_{i}^{-}s_{i}^{-}}{x_{ik}}}{\phi +\varepsilon^{+}\left(\sum_{r=1}^{s}\frac{w_{r}^{+}s_{r}^{+}}{y_{rk}}+\sum_{p=1}^{q}\frac{w_{p}^{u-}s_{p}^{u-}}{u_{pk}}\right)}$$

(2) $$s.t\left\{\begin{array}{c} \sum_{j=1,j\neq k}^{n}\lambda_{j}x_{ij}+s_{i}^{-}=\theta x_{ik}\;,i=1,\cdots ,m\\ \sum_{j=1,j\neq k}^{n}\lambda_{j}y_{rj}-s_{r}^{+}=\phi y_{\gamma k}\;,r=1,\cdots ,s\\ \sum_{j=1,j\neq k}^{n}\lambda_{j}u_{pj}+s_{p}^{+}=\phi u_{pk}\;,p=1,\cdots ,q\\ \lambda_{j}\geq 0,s_{i}^{-},s_{r}^{+},s_{p}^{+}\geq 0 \end{array}\right.$$

where, $r^{*}$ represents the EE of animal husbandry, k represents the number of decision units; $x_{ik},y_{rk},u_{pk}$ represent the input, expected output and unexpected output of the k decision unit, respectively; $s_{i}^{-},s_{r}^{+},s_{p}^{u-}$ relaxation variables represent input, expected output and unexpected output, respectively; $w_{i}^{-},w_{r}^{+},w_{p}^{u-}$ indicate the relative importance of input indicators, expected outputs, and non-expected output indicators; θ and φ represent the planning parameters of the radial part; ε represents the degree of combination of the key parameters, namely radial and non-radial relaxation, satisfied 0≤ε≤1.

#### 3.2.3. Control Variable Selection and Measurement

To reduce bias caused by missing variables and improve research accuracy, this paper uses the traditional STIRPAT model as a reference, selects control variables from population, economic development, and technical levels, and expands the STIRPAT model by introducing feed resource productivity and land resource as other factors affecting the EE of animal husbandry, as shown in Table 3.

Population considerations include the cultural level of the rural workforce and the rate of urbanization. The improvement in the cultural level of the rural labor force indicates that the higher animal husbandry practitioners’ environmental awareness and acceptance of emission reduction technologies, the more accurate and reasonable treatment of livestock and poultry manure will be promoted, and the EE will improve. The increase in urbanization rate indicates that the transition from agricultural to urban population is accelerating, which leads to a decrease in livestock breeding labor force and an increase in demand for livestock products, promoting large-scale animal husbandry production and indirectly affecting animal husbandry EE [20].

Economic development considerations primarily comprise the amount of economic development of animal husbandry and its fraction of total output value. Shafik and Bandyopadhyay (1992) [39] proposed the environmental Kuznets curve theory, believing that as the economy continues to develop, the environment will deteriorate and eventually improve. As a result, there may be an inverse U-shaped link between pollution from animal agriculture and economic progress. When the proportion of output value of animal husbandry is higher, it indicates that it has become the local core industry, and the local government is more likely to promote the sustainable development of animal husbandry through policy support and resource investment.

The technical level comprises animal husbandry mechanization as well as green innovation technology. Science and technology have a significant role in the modernization of animal husbandry. On the one hand, science and technology can lower production costs and increase economic advantages by enhancing feeding and management practices and increasing cattle and poultry production rates [40]. Green farming technology, on the other hand, can treat livestock and poultry waste in a unique way while also reducing environmental pollution.

Among the expanding elements, feed resource productivity is a critical fundamental assurance for animal husbandry. On the one hand, it encourages animal husbandry to expand production scales and increase animal product output; on the other hand, energy consumption in feed production and excessive feed use will result in resource waste and pollution emissions, further affecting animal husbandry’s EE [18]. Land resources can provide a location for the consumption of cattle and poultry waste, encourage the creation of ecological cycle breeding models, and influence the EE of animal husbandry to some level.

### 3.3. Research Methods

#### 3.3.1. Benchmark Model

Research and theoretical analysis indicate that the effect of environmental control on the energy efficiency of animal husbandry may be non-linear. Given that the influence of environmental rules on industry evolves dynamically, it is more pragmatic to employ smooth curves for nonlinear fitting in this research. Simultaneously, prior research indicates that the use of cubic terms enhances data fitting [41]; hence, this article employs higher-order variables to construct a benchmark model, as outlined in Equation (3):

(3) $$Y_{it}=\gamma_{0}+\gamma_{1}X_{it}+\gamma_{2}ER_{it}+\gamma_{3}ER_{it}^{2}+\gamma_{4}ER_{it}^{3}+\mu_{i}+\nu_{t}+\varepsilon_{it}$$

In the equation: $Y_{it}$ is the animal husbandry EE of i province in t year, $ER_{it}$ is the environmental regulation intensity of i province in t year, X is a series of control variables and $\gamma_{0}$ is a constant term, $\gamma_{2},\gamma_{3},\gamma_{4}$ are the core explanatory variable coefficients, $u_{i}$ and $v_{t}$ are the individual spatial and time effects, respectively, and $\varepsilon_{it}$ is the random disturbance term.

#### 3.3.2. Spatial Correlation Test Model

To investigate the spatial spillover impact between environmental regulation and animal husbandry EE, the Global Moran’s I index was first employed to evaluate the spatial correlation of animal husbandry EE, as given in Equation (4).

(4) $$Moran’s\;I=\frac{n\sum_{i=1}^{n}\sum_{j=1}^{n}W_{ij}(x_{i}-\bar{x})(x_{j}-\bar{x})}{\left(\sum_{i=1}^{n}\sum_{j=1}^{n}W_{ij}\right)\sum_{i=1}^{n}{\left(x_{i}-\bar{x}\right)}^{2}},(i\neq j)$$

In the equation: $x_{i}$ and $x_{j}$ are the EE of animal husbandry in i,j region, respectively. n is the number of provinces and $W_{ij}$ is the spatial weight.

#### 3.3.3. Spatial Econometric Model

The occurrence of geographical spillovers defies the notion that variables are independent of one another. To address the biased regression estimates caused by geographical dependence, a spatial econometrics model is employed. The spatial lag model (SAR), spatial error model (SEM), and spatial Durbin model (SDM) are the three most prevalent spatial measurement models. SDM is a combination expansion form of SAR and SEM that takes into account the spatial lag correlation of both independent and dependent variables. Therefore, the spatial Durbin model is created with reference to Elhorst’s (2014) [42] research, and its form is presented in Equation (5):

(5) $$Y_{it}=\theta_{0}+\rho \sum_{j=1}^{n}W_{ij}Y_{jt}+\beta X_{it}+\theta_{1}\sum_{j=1}^{n}W_{ij}ER_{jt}+\theta_{2}ER_{it}+\theta_{3}ER_{it}^{2}+\theta_{4}ER_{it}^{3}+\theta_{5}\sum_{j=1}^{n}W_{ij}X_{jt}+\mu_{i}+\upsilon_{t}+\varepsilon_{it}$$

In the equation: $Y_{it}$ is the animal husbandry EE of i province in year t, $ER_{it}$ is the environmental regulation intensity of i province in year t, X is a series of control variables, $\theta_{0}$ is a constant term, ρ, $\theta_{1}$ and $\theta_{5}$ are the spatial lag coefficients of dependent and explanatory variables, and $\theta_{2},\theta_{3},\theta_{4}$ are the core explanatory variable coefficients, $u_{i}$ and $v_{t}$ are the individual spatial and time effects, respectively, and $\varepsilon_{it}$ is the random disturbance term.

### 3.4. Data Sources

This article investigates 31 Chinese provinces (Hong Kong, Macao, and Taiwan are not included). Data on environmental regulations are sourced from the Peking University Talisman Database. The basic data for animal husbandry EE and other control variables were obtained from the China Agricultural and Rural Statistical Yearbook, China Statistical Yearbook, China Animal Husbandry and Veterinary Yearbook, National Agricultural Product Cost and Benefit Yearbook, Brick Agricultural Database, National Bureau of Statistics, and Provincial Statistical Yearbook, among others. Some missing data were supplemented using the linear interpolation method.

Table 4 provides descriptive statistics for the variables. The average EE is 0.748, which leaves plenty of opportunity for improvement overall. The mean value of environmental regulation is 6.169, with a standard error of 0.074, suggesting that regional governments’ environmental regulatory policies differ. The average value for green innovation technology in animal husbandry was 17.710, with a standard error of 1.085. The green innovation technology in different countries and years varied greatly; hence, logarithmic treatment was used for this variable.

## 4. Results

### 4.1. Temporal and Spatial Characteristics of Animal Husbandry Environmental Regulation

#### 4.1.1. Timing Characteristics

**Figure 3:**
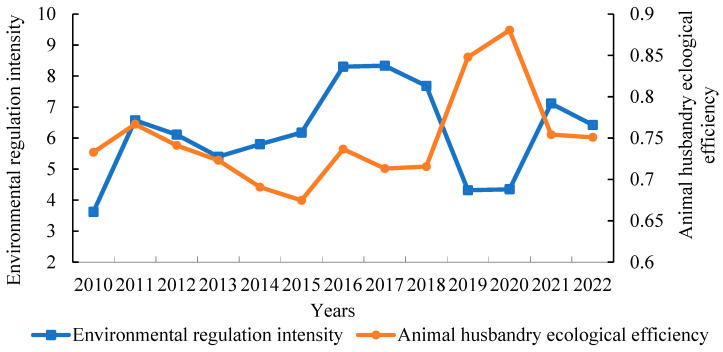
Temporal changes of EE and environmental regulation in China’s animal husbandry during the period 2010–2022. Data source: MAXDEA 8 Ultra software calculation results and “Peking University Magic” data collation.

Figure 3 shows the level of environmental control in China’s animal husbandry from 2010 to 2022.

The level of environmental control in animal husbandry in China varies. Specifically, the intensity of environmental regulations increased from 3.62 in 2010 to 6.42 in 2022, representing an increase of 77.35%, indicating that the government has gradually increased its focus on the ecological benefits of animal husbandry and promoted high-quality development of the industry.

From 2010 to 2016, the intensity of environmental regulation fluctuated, increasing from 3.62 in 2010 to 8.30 in 2016, with a small decrease from 2011 to 2013. During this period, the “Regulations on Pollution Prevention and Control of Large-scale Livestock and Poultry Farming” issued by the state in 2014 became a breakthrough in the standardization of cleaner production policies in animal husbandry. The implementation of the “Animal Husbandry Law of the People’s Republic of China” and the “Environmental Protection Law of the People’s Republic of China” in 2015 signified the successful establishment of law construction in animal husbandry. During the period 2017–2022, the severity of environmental restrictions remained consistent and declined, forming a “V-shaped” pattern. In 2017, the state conducted the second national assessment of pollution sources, while the central environmental supervision team conducted large-scale environmental protection inspection measures, and the environmental regulatory intensity of animal husbandry reached a record high of 8.33. In this period, policies mainly focused on the comprehensive utilization of manure resources, such as the “Work Points for the Resource Utilization of Livestock and Poultry Breeding Waste in 2019”. The regulatory intensity has lessened as regulations have been adjusted and optimized, indicating the government’s efforts to strike a balance between environmental protection in animal husbandry and economic development.

#### 4.1.2. Spatial Characteristics

**Figure 4:**
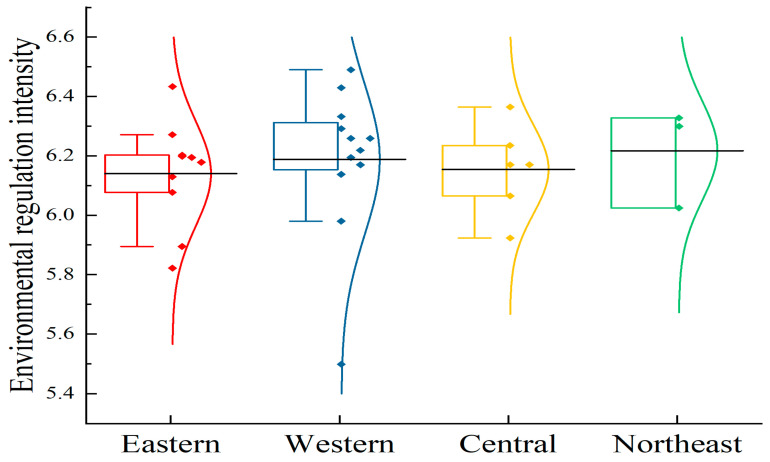
Spatial difference of environmental regulation intensity of animal husbandry in China during the period 2010–2022. Note: The black horizontal line represents the mean value.

Figure 4 shows the spatial growth of environmental legislation. China has four economic regions (The east includes Beijing, Tianjin, Hebei, Shanghai, Jiangsu, Zhejiang, Fujian, Shandong, Guangdong and Hainan; The central part includes Shanxi, Anhui, Jiangxi, Henan, Hubei and Hunan; The west includes Guangxi, Inner Mongolia, Chongqing, Sichuan, Guizhou, Yunnan, Tibet, Shaanxi, Gansu, Qinghai, Ningxia and Xinjiang; Northeast: Liaoning, Jilin, Heilongjiang): eastern, central, western, and northeastern.

The level of environmental regulation of animal agriculture in China demonstrates some imbalanced characteristics. According to the average value of each region, the intensity of environmental regulation in the northeast region (6.22), the western region (6.19), the central region (6.15), and the eastern region (6.14) exceeds the national average level (6.18), while the central and eastern regions are slightly lower. However, the mean difference is modest. In terms of fluctuation degree, the eastern region has the highest concentration of environmental regulatory intensity, although Beijing and Hainan Province have two “extreme values”. Liaoning Province (6.02) in Northeast China has a comparatively low environmental control intensity and a significant degree of data dispersion.

### 4.2. Temporal and Spatial Characteristics of Animal Husbandry EE

#### 4.2.1. Timing Characteristics

Figure 3 shows the environmental impact of animal husbandry in China from 2010 to 2022.

The EE of animal husbandry in China fluctuated upward. Specifically, the EE value increased from 0.733 in 2010 to 0.751 in 2022, indicating a slow upward trend, but the total level remains low, indicating that the ecological economic balance of animal husbandry has the potential to improve.

From 2010 to 2015, the EE of animal husbandry showed a fluctuating reduction, which was primarily due to governmental changes, technological lag, and economic pressure. During the period 2016–2022, the EE fluctuated upward, owing primarily to increased environmental awareness among breeding subjects, advancements in green production technology in animal husbandry, and the transformation and upgrading of some breeding enterprises, among which the EE decreased between 2017 and 2018. The implementation of the “one-size-fits-all environmental protection” regulation may have increased transformation pressure on the farm, while new technology has yet to completely play its role, resulting in a short-term drop in EE.

#### 4.2.2. Spatial Characteristics

**Figure 5:**
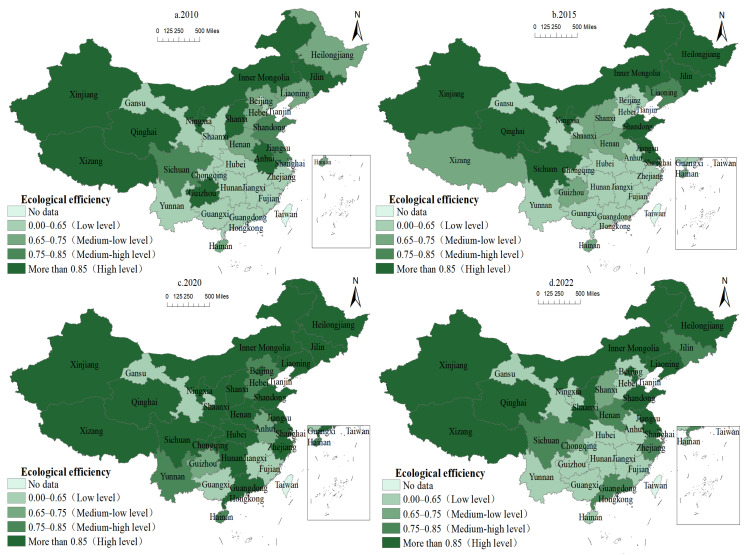
Spatial distribution of EE of animal husbandry in China from 2010 to 2022.

Figure 5 shows the regional distribution of animal husbandry EE.

The number of high-value EE regions in animal husbandry followed a “first increase, then decrease” pattern. From 2010 to 2015, the most efficient regions were northeast China and Xinjiang, Qinghai, Tibet, and Inner Mongolia in the west, whereas the majority of other regions had efficiency levels less than 0.65. In 2020, the number of regions with a high efficiency level (above 0.85) increased, accounting for 67.74% of the total, with the majority of the increase coming from eastern and central regions, and the level of EE improved significantly. In 2022, the general efficiency level has reduced compared to 2020, and Shanxi, Henan, and Sichuan in the central and western regions have withdrawn from high-efficiency zones, resulting in a lower efficiency value.

The polarization of EE in animal husbandry is strong and consistent. Between 2010 and 2022, the EE of animal husbandry in China was primarily high and low, with both exhibiting a centralized and contiguous “block” distribution pattern. The middle-low and middle-high level regions had a “scattered” distribution pattern, gradually shifting from the northeast and eastern regions to the center and western areas.

### 4.3. Impact of Environmental Regulation on EE of Animal Husbandry

#### 4.3.1. Nonlinear Impact of Environmental Regulation on EE of Animal Husbandry

In this paper, the Hausman test was performed first, and the results (H1 = 74.81 ***) showed that the fixed-effect model should be used. Second, following a thorough evaluation of individual, time-fixed, and dual-fixed models, the dual-fixed effect model was chosen. Table 5 shows the relevant results.

(1) Environmental regulation

Environmental regulation has an “N” type impact on animal husbandry EE, with inflection points at 6.322 and 9.456, indicating that as environmental regulation intensity increases, the EE of animal husbandry exhibits a “growth–decline–growth” stage feature, and Hypothesis 1 is confirmed. Under the assumption that all other conditions remain constant when the environmental regulation intensity is less than 6.322, the environmental regulation intensity grows by 1 unit and the EE of animal husbandry increases by 0.538 units. When the environmental regulation intensity was greater than 6.322 but less than 9.456, the environmental regulation intensity grew by 1 unit while the EE of animal husbandry declined by 0.071 units. When the environmental regulation intensity exceeds 9.456, increasing the environmental regulation intensity by 1 unit will result in a 0.03 unit rise in animal husbandry EE, with the positive marginal effect of environmental regulation being weak.

When the intensity of environmental regulation is between the two inflection points (6.322–9.456), enterprises are faced with more stringent carbon emission standards, the demarcation of prohibited culture areas and the improvement of industrial access thresholds (such as the implementation of the Action Plan for Water Pollution Prevention and Control). At this time, the “compliance cost” effect is dominant, and enterprises need to invest a lot of money in upgrading pollution control facilities or adjusting production structures, resulting in a surge in ecological aquaculture costs. In this stage of policy implementation, the “one-size-fits-all” phenomenon intensified the compliance pressure, squeezed the livestock production funds, and inhibited the improvement of ecological efficiency. When the severity of environmental restrictions exceeds the second inflection point (9.456), the policy turns to technology guidance and industrial upgrading support, enterprises gradually reduce compliance costs through innovative means such as cleaner production technology and waste resource utilization, and the effect of “innovation compensation” begins to appear. However, the current application degree and resource allocation efficiency of technological innovation are still limited by problems such as lagging technology promotion and insufficient capital investment, resulting in its positive effect on ecological efficiency has not been fully released.

(2) Other factors

The levels of urbanization (Urb), animal husbandry economic development (Aed), animal husbandry mechanization (Ahm), and feed resource capacity (Frq) all met the 10% significance level. Among these, the economic development level (Aed) of animal husbandry has a positive impact, and the coefficient was 0.034. Urbanization level (Urb), livestock mechanization level (Ahm) and feed resource productivity (Frq) had negative effects, and the coefficients were −3.703, −0.021 and −0.357, respectively. The reason for this is that improving animal husbandry’s economic development level can help it optimize its industrial structure, enhance resource allocation, promote the use of clean production and technology, and therefore improve its EE. On the one hand, increased urbanization has a siphon effect on young rural labor and capital, and animal husbandry slows the growth rate of pollution reduction and carbon reduction; on the other hand, producers increase animal product output in a limited resource environment to meet ever-increasing market product demand, stifling EE improvement. Improved automation levels can boost animal husbandry output, but they also increase emissions of animal husbandry pollutants, which has a negative influence on EE. High-quality feed may give ample nutrients to cattle and poultry while also improving production efficiency; nevertheless, feed production requires a large amount of land, water resources and energy (such as fertilizer, pesticides and processing energy consumption). At the same time, excessive feed use directly leads to a surge in livestock and poultry manure emissions. If the treatment technology is insufficient, it will aggravate water eutrophication and air pollution, and further inhibit the improvement of ecological efficiency.

#### 4.3.2. Spatial Spillover Effect of Environmental Regulation on EE of Animal Husbandry

(1) Spatial Autocorrelation Test

Before exploring the spatial effect of environmental control on animal husbandry EE, it is necessary to test the existence of spatial autocorrelation. As a result, the Moreland index is computed, and the relevant data are presented in Table 6. With the exception of 2020, the worldwide Moreland index of animal husbandry EE in China was positive for the remaining study years. The test of significance revealed that animal husbandry EE had a strong spatial association and agglomeration. The degree of spatial agglomeration shows a trend of “rising, stable, and declining” over time, with the decrease being greater in 2020 due to the influence of the epidemic. In terms of spatial dimension, the EE of animal husbandry in China throughout the studied period exhibited a positive spatial correlation, with certain features of high and low concentration.

(2) Model testing and selection

There is some spatial autocorrelation in the EE of animal husbandry in China, thus additional tests are carried out to determine an acceptable spatial measurement model, and the findings are reported in Table 7. First, the LM test reveals a spatial effect at the 1% significance level, implying that spatial regression is required. Second, the LR and Wald tests indicate that the spatial Durbin model (SDM) degenerates into the spatial lag model (SAR) and the spatial error model (SEM). Third, Hausman test findings indicate that a fixed effect model should be adopted; third, joint significance results indicate that both the individual fixed effect and the temporal fixed effect pass the 1% significance level test, implying that the double fixed effect should be used. Finally, based on the model testing findings, this research chooses the dual fixed effect spatial Durbin model for empirical analysis.

(3) Analysis of regression results

Table 8 shows the regional effects of environmental legislation on the energy efficiency of animal husbandry in China. The spatial autocorrelation rho coefficient of animal husbandry EE is highly positive, and the spatial matrix estimation coefficient (Wx) does not accurately reflect the geographic spillover effect of independent variables on dependent variables. Based on the work of LeSage et al. (2009) [43], this paper applies the partial differential method to the model to obtain the direct effect, indirect effect, and total effect, with the indirect effect reflecting the spatial spillover effect of environmental regulation on the EE of animal husbandry.

Environmental restriction caused an “inverted N” type spatial spillover impact on the EE of animal husbandry in nearby areas, with extreme values of 5.33 and 7.67. Hypothesis 2 was validated. That is, as the region’s environmental control intensity increases, the EE of animal husbandry in nearby areas exhibits a “decline–growth–decline” pattern. Specifically, if other conditions remain unchanged, when the intensity of environmental regulation is lower than 5.33, the coefficient is −1.962. When the environmental regulation intensity is higher than 5.33 and lower than 7.67, the coefficient is 0.312. When the environmental regulation intensity is higher than 7.67, the coefficient is −0.016.

Environmental control can affect the EE of animal husbandry in nearby areas via a spatial spillover effect, and when the degree of environmental regulation increases, specific stage features emerge. When the intensity of environmental regulation falls within a specific range (5.33–7.67), the “cohort effect” has a significant positive impact on the EE of animal husbandry in neighboring areas, which may be reflected in competition mechanisms and policy coordination. When the level of environmental control exceeds the above threshold, it has a detrimental impact on the EE of animal husbandry in nearby areas. Specifically, when the intensity of environmental regulation is less than 5.33, neighboring areas lack the motivation to improve environmental protection measures, thereby impeding EE improvement; when the intensity of environmental regulation is greater than 7.67, high-polluting enterprises in the region may relocate to neighboring areas to avoid strict environmental protection requirements, resulting in a “pollution refuge” effect in neighboring areas.

#### 4.3.3. Heterogeneity Analysis of Environmental Regulation on EE of Animal Husbandry

(1) Geographical Location Heterogeneity

The heterogeneity of the impact of environmental restrictions on animal husbandry eco-efficiency due to location differences was investigated, and the findings are presented in Table 9.

The regression results in China’s western and northeastern areas match the overall regression results. Environmental laws have an “N” type impact on the EE of animal husbandry, with extreme points in the two regions being 5.23 and 8.34, and 4.593 and 8.836, respectively. Hypothesis 3 is proven. When the intensity of environmental restriction falls between the two extremes, it has a considerable detrimental impact on the EE of animal husbandry. The government must impose stricter environmental regulations on animal husbandry producers in order to boost the two regions’ green development processes and improve animal husbandry’s EE. Simultaneously, the two extreme points in the western and northeastern regions exhibited a pronounced leftward trend relative to the national average, signifying that the threshold for the adverse impact of environmental regulation on the economic efficiency of animal husbandry in these regions was diminished. This indicates a heightened sensitivity to environmental regulation, necessitating greater caution in the formulation of such policies to ensure their scientific validity and efficacy.

The regression results in China’s eastern and central regions differed from those in the rest of the country, and environmental rules considerably enhanced animal husbandry efficiency. Specifically, when the intensity of environmental control in the eastern and central regions is between (3.73, 8.39) and (3.61, 8.44), it has a considerable favorable effect on local animal husbandry’s EE. The possible explanation is that, in comparison to the western and northeast regions, the resource and environmental carrying capacity of animal husbandry in the eastern and central regions is limited, and the increase in environmental regulation intensity forces producers to accelerate the pace of green production transition, which has a consistent positive impact.

(2) Policy time heterogeneity

In 2010, the Bulletin of the First National Pollution Source Survey identified concerns with livestock and poultry waste disposal during the livestock production process, emphasizing the gravity of animal husbandry pollution. Since then, governments at all levels have implemented a number of measures aimed at reducing pollution from animal agriculture. In 2017, the country launched its second national survey of pollution sources, and the 19th National Congress report clearly stated that resource conservation and recycling should be promoted, emphasizing the importance of environmental protection and sustainable development [5]. The animal husbandry policy has been constantly refined and altered, with the policy goal shifting from pollution control to resource utilization and green production. As a result, the heterogeneity of the impacts of environmental regulatory policies with different orientations on animal husbandry EE in 2010–2016 and 2017–2022 was investigated, and the findings are presented in Table 10.

Environmental laws had an “N” type impact on animal husbandry’s EE between 2010 and 2016, with extreme values of 5.895 and 8.596, although the effect was not significant. Environmental regulation had a large “N-type” effect on animal husbandry EE from 2017 to 2022, with extremes at 7.29 and 7.35. When the environmental regulation intensity was less than 7.29, animal husbandry EE rose as the level of environmental regulation increased. When the level of environmental control falls between (7.29–7.35), it has a detrimental impact on animal husbandry’s energy efficiency. When the intensity of environmental control exceeds 7.35, it will continue to encourage the improvement of EE, confirming Hypothesis 4.

In the first stage, the EE of animal husbandry was not significantly affected by the implementation of environmental regulation policies, indicating that in the early stage of the study, single restrictive policies such as industry standards, administrative orders, and government assessments had no significant impact on the EE of animal husbandry. In the second stage, environmental regulation has a major “N” type impact on the EE of animal husbandry, which means that various policy measures such as financial subsidies and waste resource usage have an impact on the economic and ecological advantages of animal husbandry. At the same time, the interval between extreme value points decreased from 5.895–8.596 in the first stage to 7.29–7.35 in the second stage, showing that the intensity range of environmental laws to inhibit the EE of animal husbandry gradually reduced.

## 5. Discussion

### 5.1. Temporal and Spatial Characteristics of Animal Husbandry Environmental Regulation

#### 5.1.1. Timing Characteristics

The severity of environmental regulation is primarily separated into two phases. The first stage runs from 2010 to 2016, and it displays a fluctuating upward tendency. The Regulations on Pollution Prevention and Control of Large-scale Livestock and Poultry Farming in 2014 may be the first agricultural environmental protection laws and regulations, after which the state implemented a series of cleaner production policies [5]. A system of laws and regulations governing animal husbandry has gradually developed. The second stage runs from 2017 to 2022, and the strength of environmental rules is steady and reducing. At this point, the green environmental regulating policy for animal husbandry was in a stable period, which increased the integration of animal husbandry production management mode and green technology research and development, as well as the policy’s practicability and applicability. Wei et al. (2021) [12] and Xiong (2022) [44] provide support for this finding.

#### 5.1.2. Spatial Characteristics

The intensity of environmental control in the northeast and western regions is higher than the national average, while the central and eastern regions are slightly lower, but the difference is small. Northeast China, in particular, is a promising area for animal husbandry growth, with apparent competitive advantages. To some extent, the government has strengthened its support for green production methods such as planting and breeding in combination, as well as the agriculture–animal husbandry cycle. The western regions of Gansu, Xinjiang, and Qinghai are important pastoral areas in China. Environmental regulation has been enhanced to improve traditional extensive production and management methods while also promoting sustainable development of animal husbandry, so it is slightly more intense than the national average.

### 5.2. Temporal and Spatial Characteristics of Animal Husbandry EE

#### 5.2.1. Timing Characteristics

EE in animal husbandry showed a variable drop from 2010 to 2015. The possible explanations are as follows: On the one hand, the scale of animal husbandry continued to grow, but production efficiency was not significantly improved, and resource utilization was low; on the other hand, breeding subjects have low environmental awareness and ignore ecological benefits, reducing EE. During the period 2016–2022, the efficiency value increased, which could be attributed to the government’s increased emphasis on clean animal husbandry production, which included measures such as demarcating prohibited breeding areas, collecting sewage treatment fees, and strengthening certification management of green animal products to promote the improvement of animal husbandry EE [5,44].

#### 5.2.2. Spatial Characteristics

The high-value area of EE for animal husbandry is primarily centered in the northeast, eastern, and western pastoral districts. Northeast China offers abundant feed supplies, a solid basis for animal husbandry development, a high environmental carrying capacity, and a strong resilience for ecological and efficient animal husbandry development. The majority of the eastern regions are economically developed coastal areas, with producers having more advanced manure treatment technology, equipment, and experience, as well as a faster ecological green transformation process in animal husbandry [3]. Western pastoral areas (Inner Mongolia, Xinjiang, Tibet, Qinghai, and Gansu) with abundant production means and resources experience less ecological pressure [3,23], and the EE is comparatively high.

### 5.3. Impact of Environmental Regulations on EE of Animal Husbandry

#### 5.3.1. Nonlinear Impact of Environmental Regulation on EE of Animal Husbandry

The influence of environmental regulation on the EE of animal husbandry showed an “N” type change trend, with inflection points at 6.322 and 9.456, indicating that as the severity of environmental regulation increased, the EE of animal husbandry showed a “growth–decline–growth” cycle. This finding differs from prior research on the positive and negative linear effects of environmental regulation on animal husbandry or agricultural green production [18] or the “U-shaped” and “inverted U-shaped” nonlinear effects [11,22].

During the period 2010–2015, environmental regulation was at a low-intensity stage (below 6.322), with the promulgation of relevant policy documents such as the Regulations on Pollution Prevention and Control of Large-scale Livestock Farming and Opinions on Accelerating the Development of Modern Animal Husbandry encouraging the initial clean production of animal husbandry. In other words, the production process can be environmentally friendly by improving the breeding process, reducing pollution emissions, and improving the efficiency of resource utilization. Animal husbandry producers reduce the scale of breeding and adjust inefficient production methods to meet the requirements of regulatory authorities. At this point, environmental governance costs (pollution treatment equipment investment, process adjustment costs, etc.) are lower, and EE has improved [45]. During the period 2016–2018, environmental regulations were at a medium intensity level (6.322–9.456), and a number of actions were adopted in various regions to tighten carbon emission limits, delimit prohibited farming areas, and raise industrial access barriers. Simultaneously, the situation of “environmental protection is one-size-fits-all” was highlighted, and high ecological farming expenses limited output finances. It has a negative impact on the stable growth of the animal husbandry economy as well as EE. During the period 2019–2022, the intensity of environmental regulation has not reached the second turning point of 9.456, but is still in the medium and low-intensity range, and it is continuously improving. At this point, environmental regulating regulations like the Opinions on Promoting High-quality Development of Animal Husbandry (2020) will gradually release the effect of “innovation compensation” through technical guidance and industrial upgrading support [11]. For example, the utilization of waste resources (such as biogas power generation) and the application of intelligent farming technology have reduced part of the compliance cost, but the positive impact of lagging technology promotion and insufficient capital investment has not fully offset the management cost (such as carbon emission reduction equipment procurement and environmental protection facilities maintenance), and the beneficial influence on EE is still building up.

#### 5.3.2. Spatial Spillover Effects of Environmental Regulations on Animal Husbandry EE

Environmental regulation had an “inverted N” spatial spillover effect on the EE of animal husbandry in nearby areas, with extreme points at 5.33 and 7.67. This is comparable to the findings of Peng (2020) [46] and Zhou et al. (2024) [47], who investigated the relationship between environmental policy and green performance. However, the “group effect” and “demonstration effect” in this paper also play a positive role, so the increase in environmental regulation intensity in the region, the EE of animal husbandry in the neighboring region presents a phased feature of “first decline–then increase–then decline”. When the intensity of environmental regulation is low (less than 5.33), the environmental governance cost in the region is low, and capital and resource elements in the neighboring region may be transferred to avoid the higher environmental regulation cost, resulting in a decrease in the economic benefits of animal husbandry in the neighboring region, and thus the EE. When environmental rules become more stringent (5.33–7.67), governments in nearby regions may use an “upward competition” strategy to prevent falling behind their own regions in environmental governance. At the same time, this region’s advanced experience technology may have a “demonstration effect” on nearby locations, thereby improving EE. However, high-intensity environmental regulations (above 7.67) may transform adjacent areas into “pollution havens” [20,46]. Specifically, during the period 2016–2018, when the intensity of environmental regulations is higher than 7.67, some high-polluting enterprises may choose to relocate to neighboring areas with looser environmental regulations, which not only intensifies environmental pollution in neighboring areas but also leads to EE decline.

#### 5.3.3. Heterogeneity Analysis of Environmental Regulation on EE of Animal Husbandry

Geographical location and policy time heterogeneity influence the impact of environmental regulations on animal husbandry EE.

Environmental control is important in encouraging the EE of animal husbandry in eastern and central China; however, it has an N-type relationship of “first increase, then decrease, and then increase” in northeast and western China. The eastern region has a high degree of economic development, reasonably mature green technology, effective execution of environmental regulating legislation, and a rapid pace of green transformation in animal husbandry. The central region is an important animal husbandry area, but due to limited resources and environmental carrying capacity, it must expedite the promotion of ecological farming in order to meet environmental protection standards. Thus, environmental control has a considerable positive impact on the eastern and central regions. This contradicts the findings of Wei and Li (2024) [48], and Liu et al. (2022) [5]. One possible explanation is that the explained variable in this work is the EE of animal husbandry, whereas the explained variable in the other two studies is the pollutant emission of livestock and poultry raising.

During the period 2010–2016, environmental rules had no substantial impact on animal husbandry EE; however, from 2017 to 2022, the impact was N-type, with extreme peaks of 7.29 and 7.35. One possible explanation is that in the early stages of the research, policy implementation is low, policy tools are relatively simple, there is a lack of effective incentive and constraint mechanisms to promote green development of animal husbandry, and there are certain restrictions on green production technology [49], and the effect of environmental regulation policies is not significant. Following the 19th National Congress, the state prioritizes resource utilization of livestock and poultry waste [12]. This includes financial subsidies, technology promotion, standard formulation, and other measures to enrich and diversify policy tools, as well as strengthen implementation. Environmental regulations have a significant impact on the EE of animal husbandry.

### 5.4. Significance of Environmental Regulation Adjustment for Animal Husbandry

China’s animal husbandry environmental regulation policy has evolved from a singular policy tool to a multifaceted one. In the early stages, the policy tools were relatively simple, and implementation was limited, therefore the environmental regulation effect was small. However, with the ongoing development of policy tools such as financial subsidies, technology promotion, standard setting, and other measures, as well as significant enforcement, the impact of environmental regulations on animal husbandry EE has grown significantly. Other nations can benefit from China’s experience and encourage green animal husbandry by improving policy instruments, enhancing enforcement, and developing a strong regulatory structure, resulting in coordinated economic, social, and environmental development.

### 5.5. Limitations

This work contributes to the existing research, although there are certain constraints to consider. First, grass-roots governments have a significant influence on the behavior of animal husbandry farmers, and environmental rules imposed by township governments have a direct impact on the production behaviors of animal husbandry practitioners. In the future, grass-roots government data can be collected further, and the influence of environmental rules on the EE of animal husbandry can be validated from the micro level. Second, the current conclusions are based on macro panel data, and lack of exploration of typical areas (such as ecologically fragile areas and intensive farming areas). In the future, representative samples can be selected and case studies can be used to trace how the dynamic characteristics of environmental regulations specifically affect the transformation of aquaculture structure and the path of clean technology adoption.

## 6. Conclusions

This paper investigates the nonlinear effects of environmental regulations on the EE of animal husbandry and the spatial spillover effects on neighboring areas during the period 2010–2022. The conclusions are as follows:

(1) The intensity of environmental control fluctuates upward, with the northeast and western regions having slightly greater levels than the center and eastern regions, indicating an imbalance. The total EE of animal husbandry in China is rising in fluctuation, but the overall level remains low (average value was 0.75). The high-efficiency zones are concentrated in the east and west.

(2) Environmental regulation has an N-type nonlinear effect on the EE of animal husbandry, with inflection points at 6.322 and 9.456. China’s environmental regulation intensity is at an inflection point, and the impact of “innovation compensation” is still mounting. In terms of spatial spillover effect, environmental regulation has an “inverted N” type phased impact on the EE of animal husbandry in nearby areas, with extreme points at 5.33 and 7.67.

(3) In terms of geographical heterogeneity, environmental control greatly increased the EE of animal husbandry in the eastern and central regions. The EE in the western and northeastern regions revealed N-type features. In terms of policy temporal heterogeneity, between 2017 and 2022, the influence of environmental restrictions on animal husbandry EE was N-type. Between 2010 and 2016, environmental policies had no substantial impact on animal husbandry EE.

In view of the above findings, the following policy recommendations are made: Firstly, optimize ecological protection through redlines, emission-reduction innovations, and dynamic assessments, tailoring region-specific green transitions while avoiding one-size-fits-all policies. Secondly, strengthen cross-regional environmental collaboration, reduce eco-protection disparities, facilitate technology and resource sharing, and prevent “pollution havens” to achieve ecological synergy. Finally, link ecological performance to governance evaluations, use subsidies and partnerships to incentivize waste management infrastructure, and enforce robust oversight to ensure policy effectiveness.

## Figures and Tables

**Table 1:** Policy rating table.

Subject and Type of Policy Release	Assign	Subject and Type of Policy Release	Assign
National law	5	Provincial local regulations	3
Ministerial-level administrative regulations	4	Provincial local government regulations	2
Departmental regulations	3	Provincial and local normative (working) documents	1

**Table 3:** Selection and description of control variables of animal husbandry EE.

	Variable Name	Variable Symbol	Variable Declaration	Reference
population factor	Cultural level of rural labor force	Edu	The average years of education of the rural labor force	Liu et al. (2022) [5]
	Urbanization rate	Urb	Urban population/total population	Du et al. (2024) [20]
Economic development factor	Economic development level of animal husbandry	Aed	Gross output value of animal husbandry/people employed in animal husbandry	Shafik and Bandyopadhyay (1992) [39]
	Proportion of output value of animal husbandry	Aop	Gross output value of animal husbandry/regional gross output value	Han et al. (2020) [18]
Technical level	Mechanization level of animal husbandry	Ahm	Animal husbandry machinery general power/animal husbandry practitioners	He et al. (2023) [40]
	Animal husbandry green innovation technology	Agt	Number of patent applications for green technology in animal husbandry	Du et al. (2024) [20]
Expansion factor	Productivity of feed resources	Frp	Total annual feed production	Han et al. (2020) [18]
	Land resources	Lr	Arable land + available grassland area	Liu et al. (2022) [5]

**Table 4:** Definition of variables and descriptive statistics.

Variable Name	Variable Symbol	Observed Quantity	Mean Value	Standard Error	Minimum Value	Maximum Value
Animal husbandry EE	AEC	403	0.748	0.013	0.206	2.445
Environmental regulation	ER	403	6.169	0.074	3.211	10.632
Cultural level of rural labor force	Edu	403	8.782	0.046	4.760	10.760
Urbanization rate	Urb	403	0.585	0.007	0.227	0.896
Economic development level of animal husbandry	Aed	403	5.636	0.162	0.822	18.484
Proportion of output value of animal husbandry	Aop	403	0.052	0.002	0.001	0.140
Mechanization level of animal husbandry	Ahm	403	4.817	0.116	1.424	13.689
Animal husbandry green innovation technology	Agt	403	17.710	1.085	0	186
Productivity of feed resources	Frp	403	2.556	0.034	0	3.652
Land resources	Lr	403	6.666	0.031	4.904	7.852

**Table 5:** Results of baseline regression model.

Variable	Animal Husbandry EE		
	Coefficient	Standard Deviation	T-Value
ER	0.538 **	0.237	2.27
ER2	−0.071 *	0.036	−2.00
ER3	0.003 *	0.002	1.82
Edu	0.258	0.208	1.24
Urb	−3.703 ***	0.744	−4.98
Aed	0.034 ***	0.009	3.74
Aop	−0.223	1.245	−0.18
Ahm	−0.021 *	0.011	−1.96
Agt	0.000	0.001	0.43
Frp	−0.357 **	0.145	−2.46
Lr	0.001	0.232	0.01
Time and region fixed effects	YES		
R2	0.455		

Note: “*, **, ***” indicates a level of significance of 10%, 5%, and 1% respectively.

**Table 6:** Moran’s I statistics of EE of animal husbandry in China from 2010 to 2022.

Year	Moran’s I	Z Statistic	*p*-Value	Year	Moran’s I	Z Statistic	*p*-Value
2010	0.248	2.921	0.002	2017	0.462	4.116	0.000
2011	0.423	3.792	0.000	2018	0.433	3.881	0.000
2012	0.501	4.431	0.000	2019	0.179	1.797	0.036
2013	0.492	4.353	0.000	2020	−0.085	−0.445	0.328
2014	0.401	3.597	0.000	2021	0.281	2.632	0.004
2015	0.436	3.887	0.000	2022	0.215	2.072	0.019
2016	0.413	3.694	0.000				

**Table 7:** Test results of spatial panel regression model.

Check Type	Statistic	Check Type	Statistic
LM (error) test	40.53 ***	LR (sdm sem) test	67.51 ***
Robust LM (error) test	2.16	Wald (sdm sem) test	100.55 ***
LM (lag) test	39.92 ***	Hausman	57.88 ***
Robust LM (lag) test	1.55	Ind	24.50 ***
LR (sdm sar) test	63.26 ***	Time	391.09 ***
Wald (sdm sar) test	105.03 ***		

Note: “***” indicates a level of significance of 1%.

**Table 8:** Regression results of spatial Durbin model of environmental regulation on EE of animal husbandry in China.

Variable	Direct Effect	Indirect Effect	Total Effect	Model Estimation Coefficient Main	Space Matrix Estimation Coefficient Wx
ER	0.631 *** (0.229)	−1.962 *** (0.667)	−1.330 ** (0.561)	0.661 *** (0.223)	−1.917 *** (0.632)
ER2	−0.084 ** (0.035)	0.312 *** (0.100)	0.227 *** (0.086)	−0.089 *** (0.034)	0.304 *** (0.095)
ER3	0.004 ** (0.002)	−0.016 *** (0.005)	−0.012 *** (0.004)	0.004 ** (0.002)	−0.015 *** (0.005)
rho				0.099 * (0.060)	
Sigma2_e				0.011 ** (0.004)	
Control variable	YES				

Note: “*, **, ***” indicates a level of significance of 10%, 5%, and 1% respectively. Robust standard error in parentheses.

**Table 9:** Results of regional heterogeneity regression.

Variable	East	Middle Part	West	Northeast
ER	0.589 * (0.342)	0.945 * (0.468)	1.452 *** (0.515)	0.845 * (0.443)
ER^2^	−0.071 (0.052)	−0.111 (0.074)	−0.224 *** (0.083)	−0.141 * (0.075)
ER^3^	0.003 (0.002)	0.004 (0.004)	0.011 ** (0.004)	0.007 * (0.004)
Control variable	YES			

Note: “*, **, ***” indicates a level of significance of 10%, 5%, and 1%, respectively. Robust standard error in parentheses.

**Table 10:** Regression results of policy heterogeneity.

Variable	2010–2016	2017–2022
ER	0.609 (0.615)	0.589 ** (0.284)
ER^2^	−0.087 (0.098)	−0.081 * (0.044)
ER^3^	0.004 (0.005)	0.004 * (0.002)
Control variable	YES	
Time and region fixed effects	YES	
R^2^	0.191	0.523

Note: “*, **” indicates a level of significance of 10%, 5%, respectively. Robust standard error in parentheses.

## Data Availability

The data presented in this study are available on request from the corresponding author.
